# MIXED METHODS INTERVENTION DEVELOPMENT OF A TELEREHABILITATION PROGRAM FOR OLDER VETERANS

**DOI:** 10.1093/geroni/igae098.0709

**Published:** 2024-12-31

**Authors:** Michelle Rauzi, Jennifer Stevens-Lapsley

**Affiliations:** Department of Veterans Affairs, Aurora, Colorado, United States; University of Colorado Anschutz Medical Campus, Aurora, Colorado, United States

## Abstract

Mixed methods approaches add value during the development of complex interventions, increasing chances of program success. As such, we employed multi-stage mixed methods study designs to develop a MultiComponent TeleRehabilitation (MCTR) program for older Veterans with multiple chronic conditions. The four stages included 1) exploratory sequential, 2) explanatory sequential, 3) embedded convergent, and 4) exploratory sequential designs. Results from stage 1 provided valuable information to support the program’s initial implementation and evaluation. Stage 2 results supported the program’s feasibility for Veterans ≥50 years with at least 3 medical conditions. Stage 2 also provided key insights to improve recruitment and enrollment procedures, adapt the intervention, and modify technology integration. The resulting adaptations were implemented and evaluated during a randomized feasibility study, which involved stage 3. The primary results supported the MCTR program’s feasibility and safety in an older (≥60 years) and physically impaired population. Secondary results demonstrated some improvement of patient level outcomes including physical function. The mixed methods findings identified salient participant differences related to how and why participants chose to engage or not engage with the technological supports used during the MCTR program. These technology engagement findings were important because the higher technology engagement group showed greater improvements in physical activity. Thus, by understanding reasons why people did not engage with the technology, we can adapt the MCTR program during stage 4 to address engagement barriers and improve patient outcomes. In conclusion, using mixed methods approaches for intervention development accelerated our research and will help enhance future implementation efforts.

